# Effects of Copenhagen Adduction Exercise on Muscle Architecture and Adductor Flexibility

**DOI:** 10.3390/ijerph19116563

**Published:** 2022-05-27

**Authors:** Diego Alonso-Fernández, Rosana Fernández-Rodríguez, Yaiza Taboada-Iglesias, Águeda Gutiérrez-Sánchez

**Affiliations:** 1 Department of Special Didactics, Faculty of Education and Sport Sciences, University of Vigo, 36005 Pontevedra, Spain; rosanafernandez@uvigo.es (R.F.-R.); yaitaboada@uvigo.es (Y.T.-I.); agyra@uvigo.es (Á.G.-S.); 2 Education, Physical Activity and Health Research Group (Gies-10-DE3), Galicia Sur Health Research Institute (IIS Galicia Sur), SERGAS-UVIGO, 36208 Vigo, Spain

**Keywords:** injury, eccentric training, 2D ultrasound, muscle architecture

## Abstract

Groin injuries are one of the most prevalent in sports, especially due to the hip adductor muscles’ weakness, which is considered as a risk factor. The Copenhagen adduction exercise (CAE) has been demonstrated to increase the strength of adductor muscles, but its effects on the architectural characteristics and flexibility of the adductors has been little studied. The aim of the present study was to analyse the impact on the muscular architecture and flexibility of the adductor musculature after 8 weeks of CAE-based training and after 4 weeks of subsequent detraining. A sample of 45 active subjects (26.1 ± 2.8 years old) were randomly divided into a control group with no intervention and an experimental group with an intervention based on 8 weeks of CAE training and 4 weeks of subsequent detraining. The muscle thickness of adductors was measured before and after training and detraining using ultrasound imaging and hip abduction range with goniometry. A significant increase in muscle thickness (left leg: +17.83%, d = 1.77, *p* < 0.001//right leg: +18.38%, d = 1.82, *p* < 0.001) and adductor flexibility was found in the experimental group (left leg: +7.3%, d = 0.96, *p* < 0.05//right leg: +7.15%, d = 0.94, *p* < 0.05), and after detraining, both variables returned to their initial values. These results could indicate that CAE would be a suitable strategy to modify the architecture of the adductors and thus form part of training protocols designed for the prevention and rehabilitation of muscle injuries.

## 1. Introduction

Injury and injury prevention is one of the most studied health issues related to physical activity and sport sciences due to its consequences on performance, cessation of practice, and economic repercussions, such as sports or work-related time off. Previous studies have looked at the specificity of injuries in various areas of life [1,2,3,4].

Determining the causes of these injuries allows preventive actions and injury prevention programmes to be established. Thus, it seems essential to identify the intrinsic factors so that they can be approached with the correct physical preparation. Reviews that analyse the risk factors for musculoskeletal injury in the military include low levels of physical fitness and low levels of strength, with strong and moderate evidence linking these to injuries, respectively [5].

Flexibility deficit has been identified as a relevant factor in the occurrence of muscle injuries [6]. However, the disparity of results makes it necessary to further investigate the relationship between excess or lack of joint mobility and its involvement in injuries. Reduced values of flexibility in the quadriceps and hamstring muscles have been identified as an intrinsic factor linked to hamstring and quadriceps injuries in football players [6]. It has also been observed that adductor ruptures or tendinopathies are related to a decreased hip abduction range for these athletes [7]. However, limited mobility in ankle dorsiflexion, high hip external rotation, and low or poor flexibility, although related, indicate insufficient evidence [5]. In contrast, the range of motion in the lower limbs could be a determining factor relating to injuries in other activities, such as dance [8].

There seems to be a growing consensus that strength training is an essential component in the physical preparation of athletes and the general population, establishing multiple benefits, with an emphasis on injury prevention. Muscle weakness has been linked to an increased risk of injury. In particular, reduced strength of the hip adductors and abductors has been suggested as one of the most important intrinsic factors that may cause groin injuries [9,10].

In many sports and activities, changes of direction or accelerations are characteristic, and these are the main situations where a decrease in the strength of the adductor musculature can be the cause of an increased risk of injury [10,11]. The strength of the adductor musculature can be the result of asymmetry, muscle imbalances, or a reduction in muscle capacity.

Eccentric exercises have been shown to be of great importance in injury prevention programmes [4]. Their advantages over classical concentric contraction exercises seem to be based on their potential to improve strength, hypertrophy, and morphological changes of the musculotendinous structure [12,13,14].

The changes in muscle architecture produced by eccentric exercises have been verified using ultrasound imaging in different muscles of the lower limbs, such as the quadriceps femoris [15,16], hamstrings [17,18,19,20] and the gastrocnemius [21], highlighting the ability of ultrasound imaging to identify the structural changes in muscles following periods of training.

Different eccentric exercises that only use the athlete’s body weight have shown their importance in the modification of muscle morphology after the preventive exercise programme. For example, in hamstring strength training, the benefits of the Nordic hamstring exercise on the semitendinosus [17] and on the biceps femoris [18], the reverse Nordic exercise on the rectus anterior [22], and the heel drop on the gastrocnemius muscles [21] have all been proven.

In terms of prevention programmes that are performed on adductors, there are studies that point to the Copenhagen adduction exercise (CAE) as an important exercise in injury prevention [23]. CAE is a non-equipment eccentric exercise proposed for strengthening the adductor and abductor hip muscles to optimise the control of asymmetries [24].

However, the impact of eccentric exercises on adductor architecture has been very poorly explored in the scientific literature. To the authors’ knowledge, only Alonso-Calvete et al. [25] performed a first approach with an 8 week CAE intervention in young football players, but the evolution of its impact after a subsequent detraining period or with other types of sample subjects has never been explored. This study could not observe a significant change in the control group after including CAE in their training programme. Given the size and specific characteristics of the sample, this initial study leaves more questions than answers. Thus, the impact CAE can have on the adductor musculature of an adult, and whether its eccentricity allows it to also influence adductor flexibility remains to be observed. Such information could have relevance in the potential application of CAE in prevention and rehabilitation programmes.

Considering the importance of the adductors in sports practice and their high incidence of injury, the aim of this study was to analyse the impact on the muscular architecture and flexibility of the adductor musculature after 8 weeks of CAE-based training and after 4 weeks of subsequent detraining in healthy physically active individuals. The hypothesis is that, (a) CAE will significantly affect MT, (b) will elongate the adductor structures, and (c) the adductor structures will not maintain these changes after a period of detraining.

## 2. Materials and Methods

### 2.1. Participants

Forty-five male recreational exercisers (group fitness activities, team sports, martial arts and athletics) with no history of lower limb injury in the last 12 months and no previous eccentric training experience were randomly divided into an experimental group (EG, n = 25) and a control group (CG, n = 20). Randomisation was computer generated. The participants regularly practiced different recreational sports modalities and volunteered to participate in the study. All participants provided written informed consent prior to the protocol, and pre-training was conducted at the Faculty of Education and Sport Sciences at the University of Vigo, Pontevedra campus, Spain. Ethical approval for the study was granted by the Autonomous Ethics Committee of Research of Xunta de Galicia, Ministry of Health (Spain) following the precepts of the Declaration of Helsinki (Reference number: 2016/158).

### 2.2. Research Design

A randomised controlled trial was conducted to examine the effect of CAE training on adductor longus (AL) muscle architecture and hip mobility. The study was conducted over 13 weeks. At week 1, participants underwent a one-day baseline assessment of LA muscle architecture and hip abduction goniometry (M1). Subsequently, the groups were randomised and only the GE members underwent two familiarisation sessions to ensure correct technical execution of the CAE. Then, in the GE, 8 weeks of eccentric training based on the CAE were carried out. In week 9, the second day of architecture and goniometry assessment (M2) was performed for both groups. Participants then underwent 4 weeks of detraining avoiding any mechanical stimuli of an eccentric nature. At the end of week 13, the third assessment day (M3) was carried out for both groups. All assessments were performed at the same time of day and under the same conditions for all participants.

#### 2.2.1. Assessment of LA Architecture

The use of ultrasound has proven to be a reliable method for the assessment of muscle architectural features [26,27].

Muscle thickness (MT) has previously been assessed with this method in hamstrings [17,20], quadriceps [16,22], the gastrocnemius muscle [21], and recently in adductors [25]. Thus, MT was determined in AL from ultrasound images obtained along the longitudinal axis of the muscle belly of the dominant limb using a B-mode ultrasound scanner (frequency = 12 MHz; depth = 8 cm; field of view = 14 × 47 mm) (GE Healthcare Vivid-i, Wauwatosa, WI, USA).

The subjects underwent a 10-min rest period before the measurements. They were placed in the supine position with the leg to be assessed in hip and knee flexion, and with external rotation at the hip [25,28]. First, the pubic symphysis was located and marked as the insertion of the adductor longus. Then, the skin was covered with a conductive gel and the probe was placed longitudinally to the muscle insertion and subsequently lowered until the muscle belly appeared on the screen [29,30]. All measurements were evaluated with minimal pressure so as not to alter the accuracy of the measurements [31]. Finally, the image was frozen and the muscle thickness of the adductor longus was measured in centimetres (cm). Analysis was performed using Image J image processing software (National Institute of Health, Bethesda, ML, USA).

All the images were performed by the same experienced ultrasound technician [17,18,21,22], and shielded from the identity of the participants during the entire analysis.

#### 2.2.2. Adductor Muscles Flexibility

Flexibility deficit has been identified as a relevant factor in the occurrence of muscle injuries [6]. The use of goniometry has proven to be a reliable method to assess the flexibility of the adductor muscles [6]. The subject was placed in the supine position with the lower limbs extended and together, with the pelvis stabilised by ensuring the anterosuperior iliac spines are at the same level. The axis of the goniometer was placed on the anterior superior iliac spine of each leg.

The fixed arm was oriented to the opposite anterosuperior iliac spine and the movable arm was placed on the longitudinal axis of the femur, and oriented towards the center of the patella. From the starting position with legs together, the dominant leg was passively moved away from the midline until femoral rotation occurred, indicating the end of adductor flexibility [32,33]. A triple measurement was performed with 30 s rest between measurements and using the arithmetic mean as the final value in sexagesimal degrees.

Reliability analysis demonstrated high test-retest reliability for MT (ICC left leg = 0.92; ICC right leg = 0.94) measurements using ultrasound imaging and flexibility (ICC left leg = 0.95; ICC right leg = 0.97) measurements using goniometry.

#### 2.2.3. “Copenhagen Adduction Exercise”

**Figure 1:**
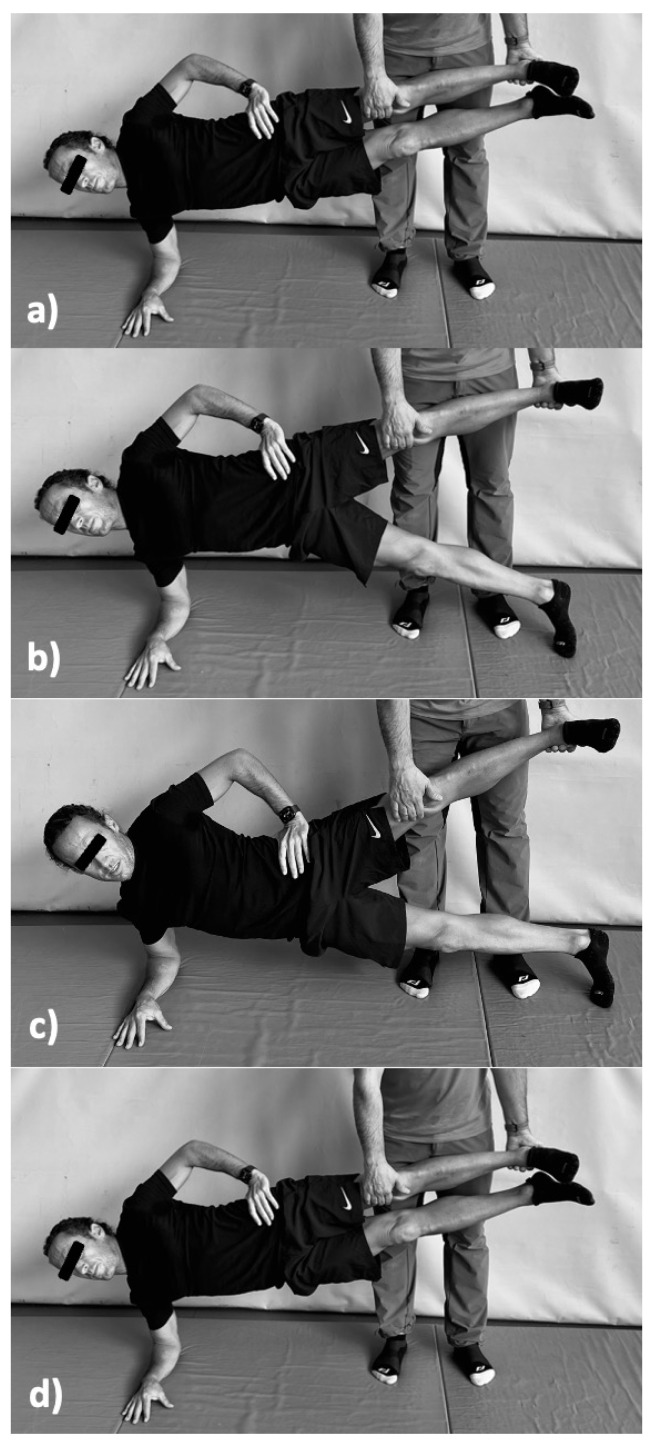
The Copenhagen adductor exercise: (**a**) starting position, (**b**) the eccentric femoral abduction phase (lower leg), (**c**) the eccentric pelvic on femoral abduction phase (upper leg), (**d**) the concentric pelvic on femoral adduction phase (lower leg) (Adapted from Schaber et al. [35]).

The CAE is defined as an exercise in which the participant assumes a side plank position, with the superior lower leg held by a partner. The participant lowers the free lower leg to the ground, producing pelvic on femoral abduction on the supported leg, then raises it to return to the start position, producing pelvic on femoral concentric adduction on the supported leg [24,34] (Figure 1).

All participants were properly instructed on the correct CAE execution technique for two days prior to training to optimise its effectiveness, and they were all supervised during all sessions of the protocol to ensure their correct performance. Based on previous scientific literature [24,35], the exercise was performed following a running tempo of 3 s in the eccentric phase and 3 s in the concentric phase. Participants were instructed and supervised at all times by the research team, and a metronome was also added to control the tempo.

### 2.3. Intervention

The GE participants completed 8 weeks of CAE-based training designed to favour a progressive assimilation of eccentric mechanical stimuli and reduce the delayed muscle soreness (DOMS) of the participants. This training programme was based on previously used programmes [23,24,25]. Each training session lasted between 10 and 20 min depending on the volume of sets and repetitions, according to the evolution of the programme (Table 1). All sessions were separated by at least 24 h and preceded by a standardised 10 min warm-up utilizing dynamic stretching and self-loading exercises.

Following the eccentric training phase, participants underwent a 4 week detraining phase avoiding any mechanical stimuli of a similar nature.

### 2.4. Statistical Analysis

Normal distribution of data was verified via the Shapiro–Wilk test and homoscedasticity via the Levene test. The Greenhouse–Geisser correction was used when the assumption of sphericity was violated (*p* < 0.05 for Mauchly’s test of sphericity). A repeated measures ANOVA was used to determine the changes induced by training in each of the muscle architecture variables (MT) and the flexibility of the adductor muscles. The within-subjects variable was time (M1-M2-M3) and the between-subjects variable was the group (EG, CG). In addition, post hoc *t*-tests with the Bonferroni correction were used to establish comparisons between variables in the different pairwise measurements (M1-M2, M1-M3 and M2-M3). Significance was set at α < 0.05 and, where necessary, Cohen’s d was provided to establish the measure of effect size. Repeatability of ultrasound and goniometry measures was evaluated using a two-way random effects intraclass correlation coefficient model, i.e., ICC [36]. The analyses were performed with SPSS 25.0 for MacOS software (IBM Corporation, Chicago, IL, USA).

## 3. Results

No significant differences in age, height or body mass were observed between the groups (*p* > *0*.05) (Table 2). The compliance rate was excellent for the EG (99%).

Table 3 sets out the results obtained in absolute values, bearing in mind the different variables measured in the sample.

### 3.1. Muscle Architecture

The eccentric training protocol with CAE affected the MT (F (2–48) = 56.27, *p* < 0.05, = 0.71). The pairwise comparisons derived from the post hoc analysis in EG showed a significant increase in the MT between M1 and M2 (left leg: t = −9.76, d = 1.77, *p* < 0.001//right leg: t = −9.96, d = 1.82, *p* < 0.001), and a significant decrease between M2 and M3 (left leg: t = 6.43, d = 1.32, *p* < 0.001//right leg: t = 6.38, d = 1.30, *p* < 0.001). For its part, the CG did not show significant changes in MT between M1 and M2 (left leg: t = −0.11, *p* > 0.05//right leg: t = 0.10, *p* > 0.05), nor between M2 and M3 (left leg: t = 0.17, *p* > 0.05//right leg: t = 0.07, *p* > 0.05).

### 3.2. Flexibility of Adductor Muscles

Hamstring muscle flexibility was affected by the CAE protocol, (F (2–48) = 47.57, *p* < 0.05, = 0.59). In the pairwise comparisons, it can be seen that the EG showed a significant increase in the degrees of hip abduction after the training protocol (left leg: t = −4.41, d = 0.96, *p* < 0.05//right leg: t = −4.39, d = 0.94, *p* < 0.05) and a significant reduction after detraining (left leg: t = 4.02, d = 0.81, *p* < *0*.05//right leg: t = 3.81, d = 0.72, *p* < 0.05). However, the CG showed no significant differences either between M1 and M2 (left leg: t = 1.17, *p* > *0*.05//right leg: t = −1.28, *p* > *0*.05) or between M2 and M3 (left leg: t = 1.59, *p* > 0.05//right leg: t = −1.43, *p* > 0.05).

## 4. Discussion

To the authors’ knowledge, this is the first study that has attempted to observe the impact that an eccentric training protocol based exclusively on CAE exercise for 8 weeks and a subsequent 4-week detraining can exert on LA muscle architecture and hamstring flexibility in healthy, active individuals. The main conclusions were that: (a) this type of training resulted in a significant increase in AL muscle thickness and hip abduction movement, and (b) that these changes were reversed after 4 weeks of detraining. The main contribution and novelty of these conclusions lies in the field of practical application, where an easily reproducible and executable exercise such as the CAE could have a place in training, rehabilitation, or prevention protocols due to its repercussions on the adductor muscle architecture.

The literature has reported that a deficit of adductor strength may be one of the main risk factors for groin pain and injury [10,37], and for an increased number of groin injuries in individuals with decreased strength in this musculature [9]. Considering this background, enhancing hip adductor muscles’ strength seems to be a valuable intervention concerning prevention strategies [38,39].

In this respect, Copenhagen adduction has been shown to be an effective exercise to significantly increase adductor strength [23,24,40,41] and prevent groin problems [42]. In this study, a significant increase (*p* < 0.001) in adductor longus muscle thickness was observed after 8 weeks of CAE training. This may be the effect on the musculature that could be related to the increase in adductor strength observed in the aforementioned studies.

Different studies have analysed architectural changes in lower limb muscles after performing protocols based on eccentric exercises [15,17,18,19,21,22], but there is only one precedent work that has studied the architectural effects of CAE on the adductor muscle architecture. Alonso-Calvete et al. [25] also obtained a significant increase in MT in the adductor longus after 8 weeks of CAE in young football players, but they could not discern whether these changes were derived solely from this intervention or from its combination with the training that the experimental subjects also performed as part of their sport. In our study, the EG performed the CAE during the experimental phase in isolation, therefore, the effects on LA architecture derive only from the intervention performed, given that the CG did not obtain any significant change in this variable. Thus, it seems that the CAE has the potential by itself to increase the MT of the LA and, therefore, to modify its architecture.

In addition, this is the first occasion where it has been possible to observe the architectural changes in MT in AL after a 4 week detraining period following the 8 week protocol based on CAE. The results show a significant reduction in MT in this period of inactivity (*p* < 0.001), which compares with previous studies in other lower limb musculatures after periods of eccentric training [18,21]. In practical terms, this fact suggests that the architectural effects derived from CAE would need, at least, an adequate exercise dosage to be maintained over time. Further studies will be needed to verify what maintenance dose athletes would need to stabilise these changes.

Another risk factor for injury suggested by the literature is the lack of muscle flexibility [6,7,43]. Specifically in the adductor musculature, Ekstrand and Gillquist [7] found links between low hip abduction ROM and adductor rupture or tendinopathy injuries in a sample of 180 first division football players. However, Witrouw et al. [6] did not find a significant relationship between the lack of flexibility of this musculature and adductor injuries, although the authors themselves caution that due to the low rate of such injuries described in their study and by the low-to-moderate power observed (52%), “one must be very careful in concluding that the flexibility of the adductor and calf muscles is not important in the development of injuries in these muscles”.

To the authors’ knowledge, this is the first work that has attempted to observe how a CAE protocol affects adductor flexibility. The results obtained show a significant increase (*p* < 0.05) in hip abduction ROM after 8 weeks of a CAE-based eccentric protocol and a decrease in this effect (*p* < 0.05) after 4 weeks of subsequent detraining. It is difficult to put this finding in perspective with previous literature as no work of a similar nature is found in adductors. However, previous research has evaluated the flexibility of other lower limb musculatures following exercise-based protocols of an eccentric nature. Alonso et al. [44] observed an increase in hip flexion ROM after 8 weeks of training based on an eccentric hamstring exercise programme and a subsequent decrease after a 4-week detraining period. Thus, it would appear that stimuli of an eccentric nature could contribute to the improvement of the mobility of these muscle structures, with the repercussions that this fact may have on injury prevention programmes.

## 5. Conclusions, Limitations and Future Research

There are limitations in the study that should be mentioned. The inclusion of more measures of eccentric adductor strength could give greater consistency to the results. For this study, only AL architecture was assessed, specifically the MT variable. Other architectural variables and other adductor muscles could show differential changes, and therefore it would not be appropriate to generalize the results. Furthermore, the sample included only male participants, a characteristic that should be taken into account when evaluating the results. Therefore, the conditioning factors that may influence this type of training to achieve lasting architectural effects remains to be explored by future studies, as does the impact of this type of training on different populations and genders of the participating athletes.

Nevertheless, CAE has been shown to be a dynamic, accessible and simple exercise to introduce into the training programmes of athletes, as it has already demonstrated its capacity to modify the architecture and flexibility of the adductor musculature. Thus, it would be interesting to continue exploring its potential for use in adductor muscle injury prevention and rehabilitation protocols in sports that experience by a high incidence of these injuries.

## Figures and Tables

**Table 1:** Training progression with CAE.

Week	No. of Sessions/Week	Sets Per Side	Repetitions	Total Repetitions Per Side/Week	Rest Between Sets
**1**	2	1	5	10	-
**2**	2	1	5	10	-
**3**	2	2	6	12	2 min
**4**	2	2	7	14	2 min
**5**	2	1	10	20	-
**6**	2	1	12	24	-
**7**	3	1	15	30	-
**8**	3	1	15	30	-

**Table 2:** Characteristics of participants (mean ± standard deviation).

Group	N	Age (Years Old)	Weight (kg)	Height (m)
**EG**	25	26.3 ± 2.9	75.3 ± 11.2	1.76 ± 0.08
**CG**	20	25.8 ± 3.1	77.7 ± 10.8	1.74 ± 0.09

**Table 3:** Changes in EG and CG variables before (M1) and after (M2) the intervention and after the period of detraining (M3) (mean ± standard deviation).

	M1 (Week 1)	M2 (Week 9)	M3 (Week 12)	% Change M1-M2	% Change M2-M3
**EG**					
**Add. Longus Muscular Thickness (cm)**					
Left Leg	0.718 ± 0.076	0.846 ± 0.081 **	0.722 ± 0.062 ##	+17.83	−14.66
Right Leg	0.702 ± 0.081	0.831 ± 0.077 **	0.713 ± 0.069 ##	+18.38	−14.20
**Hip abduction range (°)**					
Left Leg	57.6 ± 6.5	61.8 ± 6.2 *	57.9 ± 5.3 #	+7.3	−6.3
Right Leg	55.3 ± 7.5	59.2 ± 7.2 *	56.3 ± 6.2 #	+7.1	−4.9
**CG**					
**Add. Longus Muscular Thickness (cm)**					
Left Leg	0.689 ± 0.087	0.699 ± 0.077	0.672 ± 0.048	+0.44	−0.88
Right Leg	0.693 ± 0.091	0.690 ± 0.069	0.688 ± 0.059	−0.43	−0.29
**Hip abduction range (°)**					
Left Leg	53.1 ± 6.9	52.9 ± 8.2	51.6 ± 8.2	−0.3	−1.8
Right Leg	51.8 ± 7.3	52.2 ± 6.4	52.9 ± 7.1	+0.8	+1.3

*p < 0.05 vs. M1, ** *p* < 0.001 vs. M1, **#** *p* < 0.05 vs. M2, **##** *p* < 0.001 vs. M2.

## References

[1] Su B.Y., Liu M.S., De Silva P.V., Østbye T., Jin K.Z. (2021). Health-related quality of life in Chinese workers: A systematic review and meta-analysis. Glob. Health Res. Policy.

[2] Anwer S., Li H., Antwi-Afari M.F., Wong A.Y.L. (2021). Associations between physical or psychosocial risk factors and work-related musculoskeletal disorders in construction workers based on literature in the last 20 years: A systematic review. Int. J. Ind. Ergon..

[3] Sass A.C., Stang A. (2013). Population-based incidences of non-fatal injuries—Results of the German-wide telephone survey 2004. BMC Public Health.

[4] Brooks J.H.M., Fuller C.W., Kemp S.P.T., Reddin D.B. (2005). Epidemiology of injuries in English professional rugby union: Part 1 match injuries. Br. J. Sports Med..

[5] Sammito S., Hadzic V., Karakolis T., Kelly K.R., Proctor S.P., Stepens A., White G., Zimmermann W.O. (2021). Risk factors for musculoskeletal injuries in the military: A qualitative systematic review of the literature from the past two decades and a new prioritizing injury model. Mil. Med. Res..

[6] Witvrouw E., Danneels L., Asselman P., D’Have T., Cambier D. (2003). Muscle flexibility as a risk factor for developing muscle injuries in male professional soccer players. A prospective study. Am. J. Sports Med..

[7] Ekstrand J., Gillquist J. (1983). The avoidability of soccer injuries. Int. J. Sports Med..

[8] Kenny S.J., Whittaker J.L., Emery C.A. (2016). Risk factors for musculoskeletal injury in preprofessional dancers: A systematic review. Br. J. Sports Med..

[9] Kloskowska P., Morrissey D., Small C., Malliaras P., Barton C. (2016). Movement Patterns and Muscular Function Before and After Onset of Sports-Related Groin Pain: A Systematic Review with Meta-analysis. Sports Med..

[10] Whittaker J., Small C., Maffey L., Emery C.A. (2015). Risk factors for groin injury in sport: An updated systematic review. Br. J. Sports Med..

[11] Engebretsen A.H., Myklebust G., Holme I.M.K., Engebretsen L., Bahr R. (2010). Intrinsic Risk Factors for Groin Injuries among Male Soccer Players. Am. J. Sports Med..

[12] Douglas J., Pearson S., Ross A., McGuigan M. (2017). Chronic adaptations to eccentric training: A systematic review. Sports Med..

[13] LaStayo P.C., Woolf J.M., Lewek M.D., Snyder-Mackler L., Reich T., Lindstedt S.L. (2003). Eccentric muscle contractions: Their contribution to injury, prevention, rehabilitation, and sport. J. Orthop. Sports Phys. Ther..

[14] Roig M., O’Brien K., Kirk G., Murray R., McKinnon P., Shadgan B., Reid W.D. (2009). The effects of eccentric versus concentric resistance training on muscle strength and mass in healthy adults: A systematic review with meta-analysis. Br. J. Sports Med..

[15] Baroni B.M., Geremia J.M., Rodrigues R., De Acebedo F.R., Karamanidis K., Vaz M.A. (2013). Muscle architecture adaptations to knee extensor eccentric training: Rectus femoris vs vastus lateralis. Muscle Nerve.

[16] Ema R., Wakahara T., Mogi Y., Miyamoto N., Komatsu T., Kanehisa H., Kawakami Y. (2013). In vivomeasurement of human rectus femoris architecture by ultrasonography: Validity and applicability. Clin. Physiol. Funct. Imaging.

[17] Alonso-Fernandez D., Gutierrez-Sanchez Á., Garcia-Remeseiro T., Garganta R. (2018). Effects of the Nordic hamstring exercise on the architecture of the semitendinosus. Isokinet. Exerc. Sci..

[18] Alonso-Fernandez D., Docampo-Blanco P., Martinez-Fernandez J. (2018). Changes in muscle architecture of biceps femoris induced by eccentric strength training with Nordic Hamstring exercise running. Isokinet. Exerc. Sci..

[19] Bourne M.N., Duhig S.J., Timmins R.G., Williams M.D., Opar D.A., Al Najjar A., Kerr G.K., Shield A.J. (2017). Impact of the Nordic hamstring and hip extension exercises on hamstring architecture and morphology: Implications for injury prevention. Br. J. Sports Med..

[20] Timmins R.G., Shield A.J., Williams M.D., Lorenzen C., Opar D.A. (2015). Biceps femoris long head architecture: A reliability and retrospective injury study. Med. Sci. Sports Exerc..

[21] Alonso-Fernández D., Taboada-Iglesias Y., García-Remeseiro T., Gutiérrez-Sánchez A. (2019). Effects of the Functional Heel Drop Exercise on the Muscle Architecture of the Gastrocnemius. J. Sport Rehabil..

[22] Alonso-Fernandez D., Fernandez-Rodriguez R., Abalo-Núñez R. (2019). Changes in rectus femoris architecture induced by the reverse nordic hamstring exercises. J. Sports Med. Phys. Fit..

[23] Harøy J., Thorborg K., Serner A., Bjørkheim A., Rolstad L.E., Hölmich P., Bahr R., Andersen T.E. (2017). Including the Copenhagen Adduction Exercise in the FIFA 11+ Provides Missing Eccentric Hip Adduction Strength Effect in Male Soccer Players: A Randomized Controlled Trial. Am. J. Sports Med..

[24] Ishøi L., Sørensen C.N., Kaae N.M., Jørgensen L.B., Hölmich P., Serner A. (2015). Large eccentric strength increase using the Copenhagen Adduction exercise in football: A randomized controlled trial. Scand. J. Med. Sci. Sports.

[25] Alonso-Calvete A., Lorenzo-Martínez M., Padrón-Cabo A., Rey E. (2021). Effects of Copenhagen Adduction Exercise on the Architectural Characteristics of Adductors in U-17 Male Soccer Players: A Randomized Controlled Trial. Int. J. Environ. Res. Public Health.

[26] Blazevich A.J., Cannavan D., Coleman D.R., Horne S. (2007). Influence of concentric and eccentric resistance training on architectural adaptation in human quadriceps muscles. J. Appl. Physiol..

[27] Kellis E., Galanis N., Natsis K., Kapetanos G. (2009). Validity of architectural properties of the hamstring muscles: Correlation of ultrasound findings with cadaveric dissection. J. Biomech..

[28] Pesquer L., Reboul G., Silvestre A., Poussange N., Meyer P., Dallaudière B. (2015). Imaging of adductor-related groin pain. Diagn. Interv. Imaging.

[29] Davis J.A., Stringer M.D., Woodley S.J. (2011). New insights into the proximal tendons of adductor longus, adductor brevis and gracilis. Br. J. Sports Med..

[30] Campbell R. (2013). Ultrasound of the Athletic Groin. Semin. Musculoskelet. Radiol..

[31] Klimstra M., Dowling J., Durkin J.L., MacDonald M. (2007). The effect of ultrasound probe orientation on muscle architecture measurement. J. Electromyogr. Kinesiol..

[32] Greene W.B., Heckman J.D. (1994). The Clinical Measurement of Joint Motion.

[33] Tyler T.F., Nicholas S.J., Campbell R.J., McHugh M.P. (2001). The association of hip strength and flexibility with the incidence of adductor muscle strains in professional ice hockey players. Am. J. Sports Med..

[34] Serner A., Jakobsen M.D., Andersen L.L., Holmich P., Sundstrup E., Thorborg K. (2014). EMG evaluation of hip adduction exercises for soccer players: Implications for exercise selection in prevention and treatment of groin injuries. Br. J. Sports Med..

[35] Schaber M., Guiser Z., Brauer L., Jackson R., Banyasz J., Miletti R., Hassen-Miller A. (2021). The Neuromuscular Effects of the Copenhagen Adductor Exercise: A Systematic Review. Int. J. Sports Phys. Ther..

[36] Hopkins W.G. (2000). Measures of Reliability in Sports Medicine and Science. Sports Med..

[37] Ryan J., Deburca N., Mc Creesh K. (2014). Risk factors for groin/hip injuries in field-based sports: A systematic review. Br. J. Sports Med..

[38] Donaldson A., Cook J., Gabbe B., Lloyd D.G., Young W., Finch C.F. (2015). Bridging the gap between content and context: Establishing expert consensus on the content of an exercise training program to prevent lower-limb injuries. Clin. J. Sport Med..

[39] Dello Iacono A., Maffulli N., Laver L., Padulo J. (2017). Successful treatment of groin pain syndrome in a pole-vault athlete with core stability exercise. J. Sports Med. Phys. Fit..

[40] Polglass G., Burrows AWillett M. (2019). Impact of a modified progressive Copenhagen adduction exercise programme on hip adduction strength and postexercise muscle soreness in professional footballers. BMJ Open Sport Exerc. Med..

[41] Kohavi B., Beato M., Laver L., Freitas T.T., Chung L.H., Iacono A.D. (2020). Effectiveness of Field-Based Resistance Training Protocols on Hip Muscle Strength Among Young Elite Football Players. Clin. J. Sport Med..

[42] Harøy J., Clarsen B., Wiger E.G., Øyen M.G., Serner A., Thorborg K., Holmich P., Andersen T.E., Bahr R. (2018). The Adductor Strengthening Programme prevents groin problems among male football players: A cluster-randomised controlled trial. Br. J. Sports Med..

[43] Gleim G.W., McHugh M.P. (1997). Flexibility and its effects on sports injury and performance. Sports Med..

[44] Alonso-Fernandez D., Martinez-Fernandez J., Docampo-Blanco P., Fernandez-Rodriguez R. (2022). Impact of Askling L-PROTOCOL on muscle architecture, flexibility and sprint performance. Int. J. Sports Med..

